# On the Ecology and Conservation of *Sericinus montelus* (Lepidoptera: Papilionidae) – Its Threats in Xiaolongshan Forests Area (China)

**DOI:** 10.1371/journal.pone.0150833

**Published:** 2016-03-22

**Authors:** Xiushan Li, Youqing Luo, Haiyu Yang, Qingsen Yang, Josef Settele, Oliver Schweiger

**Affiliations:** 1 Key Laboratory of Forest Silviculture and Conservation of the Ministry of Education, Beijing Forestry University, 100083, Beijing, China; 2 Biodiversity Research Center, Chinese Research Academy of Environmental Science, 100012, Beijing, China; 3 Key laboratory of Oak Secondary Forest Ecosystem of Gansu Province, Research Institute of Forestry Science of Xiaolongshan Forestry Experiment Agency, 741020, Tianshui, China; 4 The Station of forests diseases and pests control and quarantine, Xiaolongshan Forestry Experiment Agency, 741020, Tianshui, China; 5 UFZ - Helmholtz Centre for Environmental Research, Department of Community Ecology, 06120 Halle, Germany; 6 iDiv, German Centre for Integrative Biodiversity Research, Halle-Jena-Leipzig, Deutscher Platz 5e, 04103 Leipzig, Germany; Institut National de la Recherche Agronomique (INRA), FRANCE

## Abstract

**Contents and Methods:**

Here we present a detailed analysis of the life history, mobility and habitat requirements of the butterfly *Sericinus montelus* on the basis of extensive field observations, experimental breeding, capture-mark- recapture (CMR) and transect surveys.

**Life History:**

We found that *S*. *montelus* has three generations per year and overwinters as pupae on shrub branches in Xiaolongshan. The adults of first generation have a peak of emergence in late April. The second generation emerges at the end of June and the third in early to middle August. Within the study region, larvae of *S*. *montelus* are monophagous on *Aristolochia contorta*. Adults fly slowly and lay eggs in clusters.

**Key Factors:**

Life tables show that natural enemies and human activities such as mowing, weeding and trampling during the egg and larval stages are key factors causing high mortality, killing up to 43% of eggs and 72% of larvae thereby limiting population growth and recovery.

**Population Ecology:**

The populations of *S*. *montelus* in Xiaolongshan have a rather patchy distribution. According to CMR data, adults fly a maximum distance of 700m within a lifespan of 6 days. The host plant *A*. *contorta*, grows along the low banks of fields, irrigation ditches and paths, and can be highly affected by agricultural activities, like mowing, weeding and herding, which impact larval survival.

**Population Maintenance:**

For *S*. *montelus* should mainly focus on reducing agricultural threats to the host plant *A*. *contorta* and on increasing habitat connectivity.

## Introduction

Habitat loss and degradation as well as climate change are considered as major drivers altering the performance, abundance and distribution of wild plants and animals [1–5]. Butterflies comprise one of the most sensitive animal taxa against human activities [6,7]. Habitat loss and fragmentation, climate change, land use, agro-chemicals and biotic interactions have numerous influences on biodiversity [8], often exemplified with butterflies [9–16].

Since the economy of China developed rapidly during the last decades, the resulting increased human activities already caused tremendous losses and fragmentation of habitats and corresponding butterfly diversity [17,11]. Along farmland margins as well as along ponds various butterflies including *Papilio xuthus* could often be seen in gardens and around the villages in the 1970’s in the suburbs of the city of Lanzhou (Gansu Province, China; Li, pers. observations). But since the 1980’s, due to higher economic rewards, agriculture has shifted to vegetable farming and agro-chemicals such as insecticides are more commonly used. This increase in agricultural intensity seems to be accompanied by drastic losses of biodiversity since many species of butterflies and other groups have vanished from the agricultural areas. Today, there is hardly a trace of these species, and the only remaining common butterfly is the cosmopolitan *Pieris rapae*, which often acts as a pest species.

One of the declining northern Chinese butterfly species is *Sericinus montelus* (Li, personal observations between 1980’s and 2015), which was selected for the present study. The butterfly occurs predominantly in ecotones of forests and agricultural areas of Northern China, such as in the Qinling Mountains. Here we analyzed species and population characteristics of *S*. *montelus* to identify the main factors in relation to their hypothesized high vulnerability to land-use change. Therefore, we performed field studies in the Gaoqiao village in the center zone of the Xiaolongshan forests area, accompanied by breeding experiments in the field and in captivity, from 2005 to 2007. In the field we used transect counting to survey the abundance of adults, larvae and host plants [18–20]. Further, we estimated population sizes, migration rates, population structure, residence times, life span and sex ratio with Capture-Mark-Recapture techniques (CMR) [21, 22, 10]. Based on field surveys, we developed life tables to analyse the key factors affecting population growth and persistence [23–27].

## Materials and Methods

### Study species

**Fig 1:**
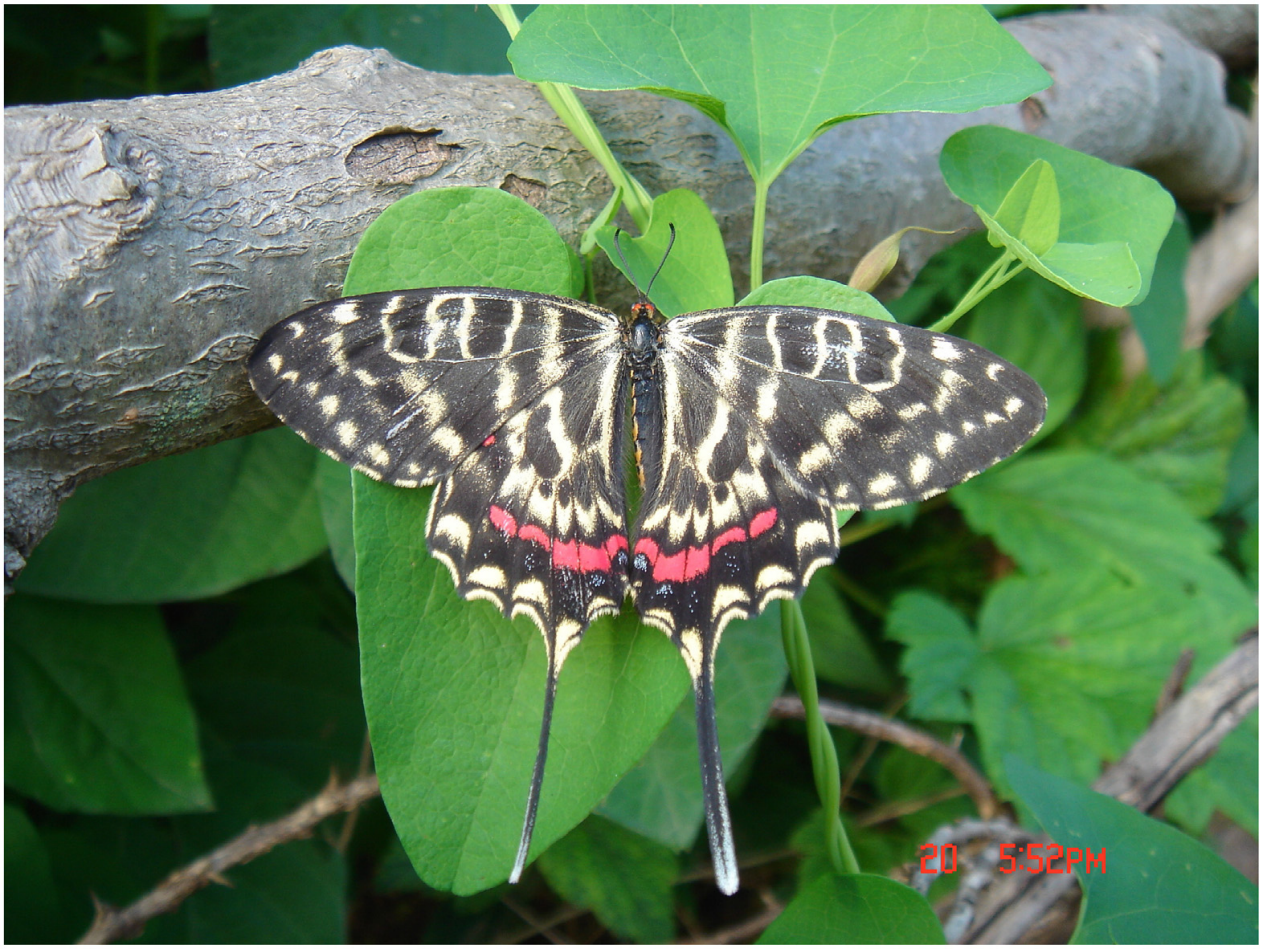
Female of *Sericinus montelus*.

**Fig 2:**
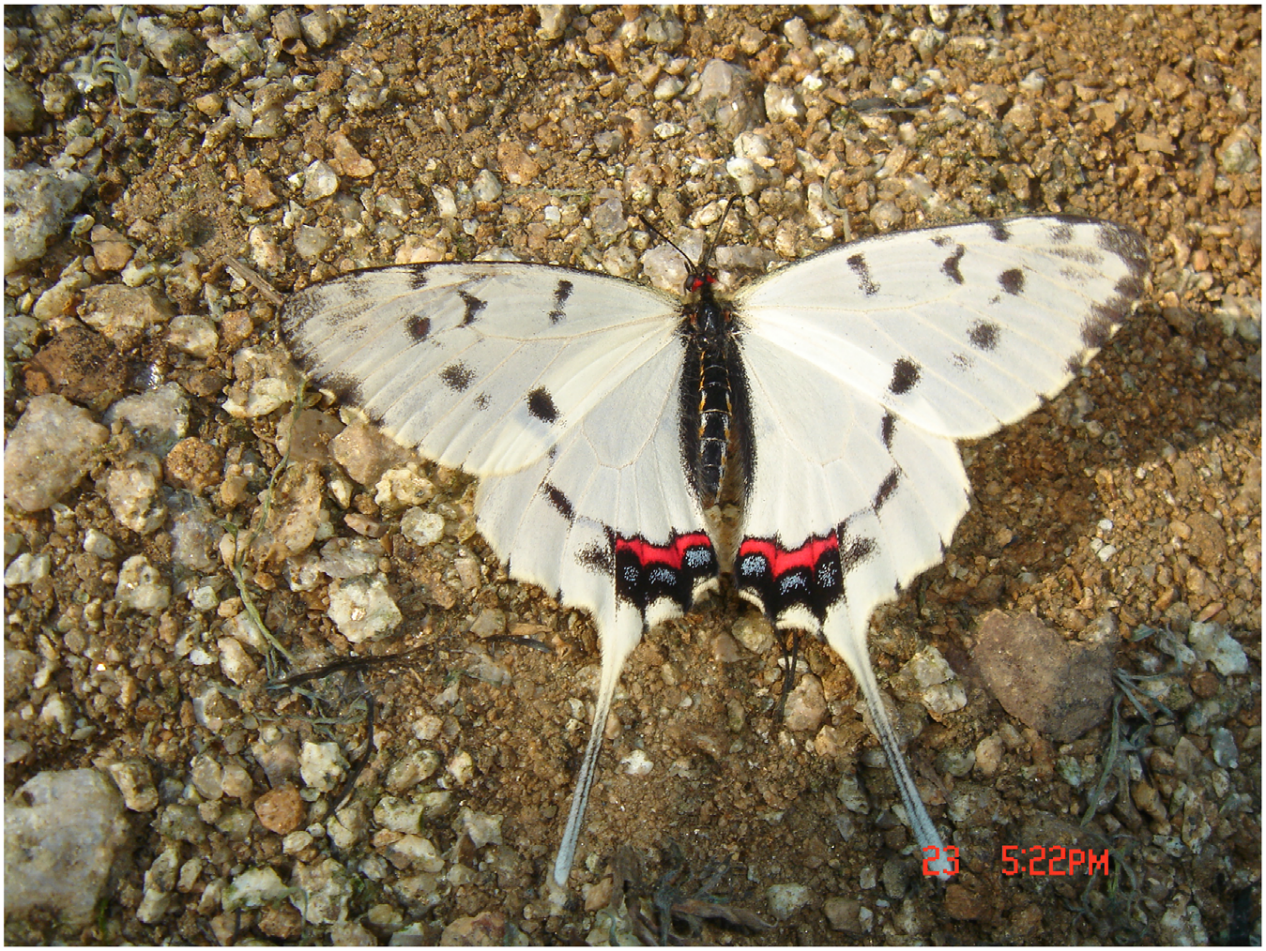
Male of *Sericinus montelus*.

*S*. *montelus* mainly occurs in the North of China, Russia, Korea and Japan [28–29]. In the Qinling Mountains it is found at the ecotone between forest and agricultural areas. In Xiaolongshan forests area (west of Qinling) it occurs in central and northern parts, including Maiji District, Huixian County, Liangdang County of Gansu province, and Taibai County in Shannxi province. Its populations are always of limited size. The adults are medium-sized with an average male wingspan of 58.0 mm and average female wingspan of 58.6 mm. The adults fly slowly, and are sexually dimorphic [28] (Figs 1 and 2). Larvae feed monophagously on *Aristolochia contorta* in Qingling Mountains, Beijing and Tianjin, while in Jiamusi (Heilongjiang province) they feed on *A*. *debilis* and *Menispermum dauricum*[30]. The development of the larvae and diapause of the pupae is influenced by temperature and photoperiod, thus the number of generations increases from the cold temperate zone to the subtropical zone [31]. In the administrative area of Jamusi *S*. *montelus* is bivoltine [30], while it has 3–4 generations in Beijing and 6 generations in Changsha and Wuhan (southern China) [31,32]. Overwintering of the pupae is initiated by changes in the photoperiod when days get shorter in autumn [33,34]. The host plant *A*. *contorta* is a perennial vine (for further details on the plant see [35]) and delineates the habitats for the butterfly. It grows along field margins and along irrigation ditches.

### Study area

**Fig 3:**
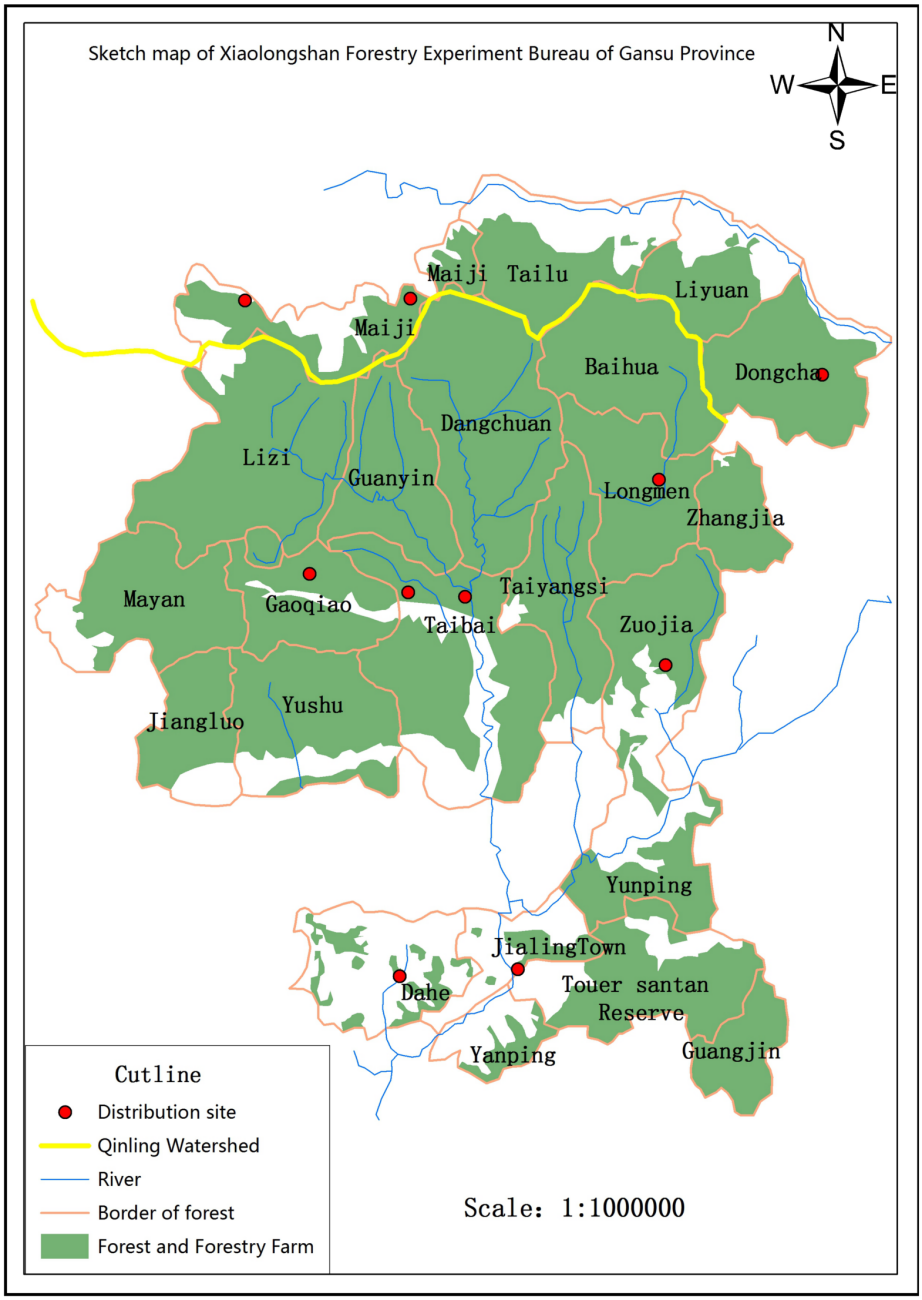
Xiaolongshan Forest and the distribution of *Sericinusmontelus*. Green: forest, red dots: site of *Sericinusmontelus*. ArcGIS 10.2 for Desktop, (Version):10.2.1. URL link: http://www.esri.com.

Field studies and captivity investigations were conducted in Gaoqiao village within the area of the Gaoqiao Forestry Farm, centrally located in the Xiaolongshan forests, on the southern slope of the western Qinling Mountains (Fig 3), with an average slope of 35°-40°. This region is located in the warm-temperate zone with a semi-humid climate, an average annual temperature of 12°C, annual precipitation of more than 750mm and 220 frost-free days. Deciduous broad-leaved forest dominates the vegetation. At the lowest ditch it is a gentle incline with farmland and an ecotone between agriculture and forestry [36]. Before 2010, farmers mainly plant wheat and corn but occasionally harvest other minor forest products such as bamboo, Chinese chestnuts and mushrooms. After 2010, due to higher economic gains compared to standard crops, more and more farmers established plant nurseries with intensive use of pesticides.

Ethics Statement: No specific permissions were required for these locations/activities. Because *Sericinus montelus* is a common species, it is not listed for specific protection. Its host plant is *Aristolochia contorta*. It grows along field margins, irrigation ditches and road margins. These places are normal public area. So it does not require specific permissions.

### Observations on biology and behaviour

Our research combined field investigations and captivity breeding. We started our field observations in late April. When female adults laid eggs on the stems of the host plants, we recorded the date of egg-laying, number of eggs, and the number of larvae hatched from the eggs. During adult flight, the behavior of flower visitation, mating and egg laying, as well as larvae predation by natural enemies were observed in the field.

Feeding and moulting of larvae were observed in experimental plots. After incubation, about 20–30 larvae were taken indoors for rearing. We used vernier callipers to measure body length and head width of larvae. Larval development was observed over all stages.

In order to know the key factor which dominates population fluctuations (and thus potentially also decline), we have done the life table research under field conditions. We used two methods: a stage-specific life table for all the three generations in 2006 [37,38], where we permanently stayed in the area for continuous observations from April to September. In 2007 we stayed for 15 to 20 days each month from April to September, thus we only could investigate a time-specific life table [23].

Mortality of eggs and larvae in the field. From late April, when overwintering adults started to lay eggs, we searched for eggs on the host plants in the field. We labelled these host plants, and recorded the egg laying date and the number of eggs. After the larvae hatched from the eggs, we labelled them in the first instar. In total 36 egg clusters or larval groups were observed continuously until pupation. Later we observed the predation by natural enemies and the failure in hatching. Observations were repeated every one to three days from April to September to calculate larval survival rates for different instars which allowed us to identify the reasons for mortality until the larvae pupated. We defined the following mortality classes: (i) ‘egg mortality due to natural enemies’ when empty eggs or natural enemy predation were observed; (ii) ‘egg mortality due to unknown reasons’ when larvae did not hatch for 20 to 30 days; (iii) ‘larval mortality because of drying up’ when dead dry larvae were found on the leaves of the host plants; (iv) ‘larval mortality because of pathogens’ when dead soft-bodied larvae were found on the host plants; (v) ‘larval mortality due to natural enemies’ when larvae disappeared from the host plants (usually larvae do not leave their hosts before the third instar, but small larvae can be swept away by heavy rain); (vi) ‘larval mortality before pupation’ when mature larvae were not able to accomplish pupation; (vii) ‘larval mortality due to human activity’ when host plants with larvae were destroyed by mowing, weeding, grazing or trampling [26].

Mortality of pupae. We gathered mature larvae, collected in the field or from indoor breeding, and let them pupate in a breeding area which also contained the host plant *A*. *contorta*. Larvae pupated after several days. All pupae were kept until eclosion. We recorded eclosion numbers and calculated mortality rates. From these rates we could infer mortality of pupae because of parasitoids and pathogens. In the field many more factors may contribute to pupal mortality such as birds or other enemies as well as direct or indirect destruction by livestock or human activity, especially during overwintering.

Key factor analysis from life table was performed according to: Ki = lg(lxi / lxi+1). K is the general K-value; K1 is the K-value of natural enemies; K2 is the K-value of failure of hatching; K3 is the K-value of human activity; K4 is the K-value of pathogens and insolation. K5 is death before or during pupation. When *Ki* has identical dynamics as *K*, and at the same time has the highest contribution to K, it is defined as the key factor [23].

**Fig 4:**
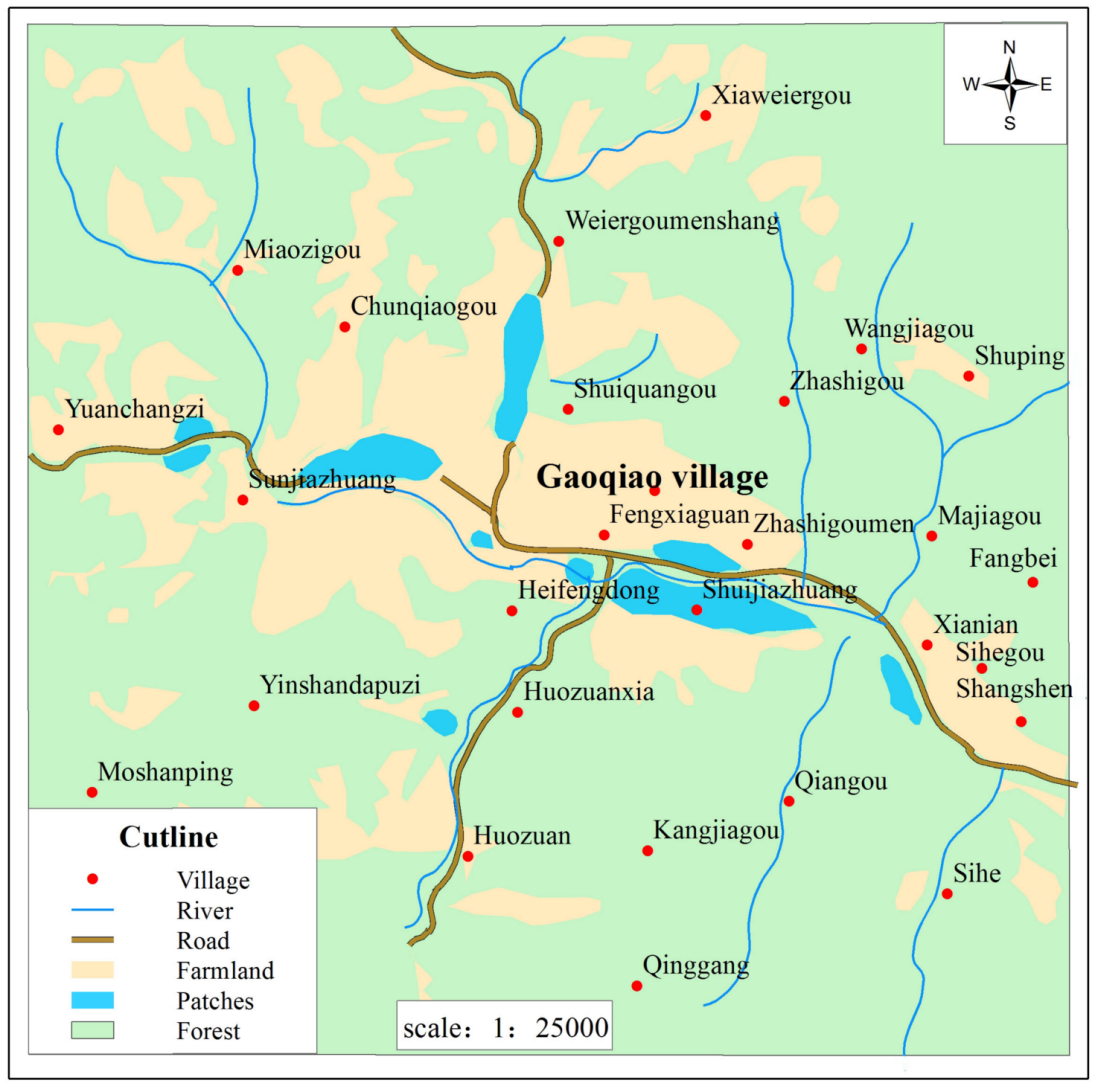
Contour map and geographic setting of habitat patches of *Sericinus montelus* in Gaoqiao village. Blue: suitable habitat patches, Green: forest. Light yellow: agricultural area. ArcGIS 10.2 for Desktop, (Version:10.2.1); URL link: http://www.esri.com.

Capture-mark-recapture (CMR) studies. Capture-mark-recapture was conducted around the Gaoqiao village, all potential habitat patches were identified by an intensive inventory. We walked through each patch; whenever there were host plants, adults or larvae present, we designated it as a potentially suitable patch. We identified three larger patches and six small patches (Fig 4). Because we were not able to detect adults in the 6 small patches, CMR could only be conducted in the three larger ones from June to August 2006, and from May to July 2007 by one or two persons. Adults were marked with a unique number using a red permanent mark pen on the underside of the left front wing and were released immediately afterwards. We recorded the number, sex, date and capture locations for further analysis [22]. When an adult was recaptured more than once on the same day, it was not considered as a recapture event. Each patch was visited once at intervals of 1–3 days for 2-hours. The CMR data were obtained for three generations: the second and third generation in 2006, and the second generation in 2007. Local population sizes (in pastures and linear elements) of each species were estimated using Jolly-Seber models as implemented in the POPAN module [39] in the Program MARK.

Sample counting and transect surveys for larval habitat requirements. To assess host plant and larval density, we used sample surveys and spot counting. Host plants were mainly distributed on the open soil patches in fields or along paths and irrigation ditches. Within these sites we established 11 sample plots of 100m×2m each. Plots were investigated once during the second to forth larval instar on all observed individual host plants *A*. *contorta* plants and *S*. *montelus* larvae were counted [27]. We calculated the density of *A*. *contorta* per sample, the percentage of host plants with larvae and average larval density, the latter as the total amount of larvae per total amount of *A*. *contorta*.

For comparison, we also walked in the shrubs or forests near the patches using transects with lengths of 400 m to 3000 m and counted the number of host plants and larvae.

## Results

### Life history

*S*. *montelus* has three generations per year in Xiaolongshan forests area. It overwinters as pupa with spring eclosion in the middle of April. The peak period of first generation of adults occurs in late April. Mating was observed on the day of eclosion and eggs have been laid on the same day or the next one. The larvae start to hatch at the end of April and can be found for about one month. The first mature larvae pupate in the first ten days of June. The peak period of the second generation of adults is from the middle to the end of June. The third generation of adults peaks between the last ten days of July and the first ten days of August. Their life history is summarized in Table 1.

**Table 1:** Life history of *Sericinus montelus*. Time: 2006–2007 Place: Gaoqiao Forestry Farm.

Stage	April			May			June			July			August			Semptember			October to next Year March		
	E	M	L	E	M	L	E	M	L	E	M	L	E	M	L	E	M	L	E	M	L
Pupae	☉	☉	☉	☉																	
Adults	⊕	⊕	⊕*	⊕	⊕																
eggs	●	●	●	●	●																
Larvae		―	―	―	―	―	―														
Pupae					☉	☉	☉	☉	☉												
Adults						⊕	⊕	⊕*	⊕	⊕											
eggs							●	●	●	●	●										
Larvae								―	―	―	―	―	―								
Pupae										☉	☉	☉	☉	☉							
Adults												⊕*	⊕	⊕							
eggs												●	●	●	●						
larvae													―	―	―	―	―				
pupae															☉	☉	☉	☉	☉	☉	☉

* Peak period of adults

### Behaviour and Characteristics of different stages

According to observations in the feeding cage (3 females and 5 males), the peak time of adult eclosion is from 10:30 to 13:00. The newly emerged adults excrete pink liquids. Wing expansion is completed within 20 minutes. About one hour elapses from eclosion to flight and males move around earlier than females. They can mate on the day of female eclosion, and mating lasts for about 15 minutes (3 females and 5 males have been kept in captivity, with 3 matings observed). Before mating, the males seek for the females. After mating females locate the host plant *A*. *contorta* and lay eggs. Adults fly slowly and flight distances are less than 700 meters (however, the number of movements detected is small, and this means that our estimates of flying distances are clearly an underestimation). Adults of the first generation lay eggs in spring on the stems of host plants near to the ground. Second and third generation females lay eggs on the underside of leaves or higher stems of the host plant. The sex ratio of female-to-male is 1: 2.29 based on pupae breeding data.

**Fig 5:**
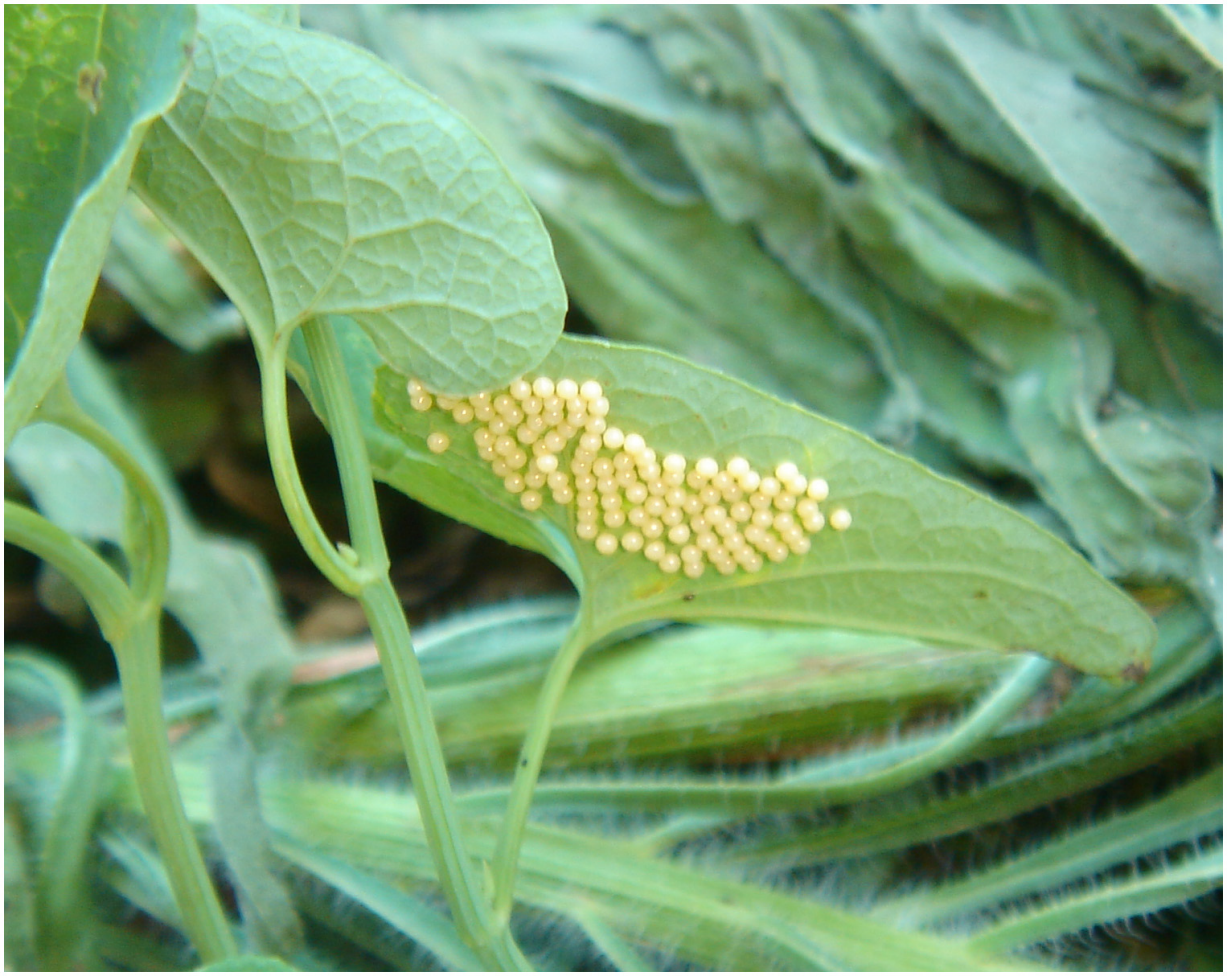
Eggs of *Sericinus montelus*.

*Eggs*: newly laid eggs are ivory but turn beige after one day (Fig 5). The diameter of the eggs is 0.8 mm. By captivity breeding we found that female can lay a maximum of two egg clusters. But if there are enough host plants, it can lay all eggs on one leaf in one cluster. The female of first generation lay 11–84 eggs with an average of 33.9±2.3 (n = 55) per cluster. It takes 7–12 days with an average of 7.5±0.68 (n = 7) days from egg laying to hatching. The second and third generation females lay 14–140 eggs per cluster with an average of 62.7±6.48 (n = 27). The egg stage lasts 5–7 (6.6±0.3; n = 7) days.

**Fig 6:**
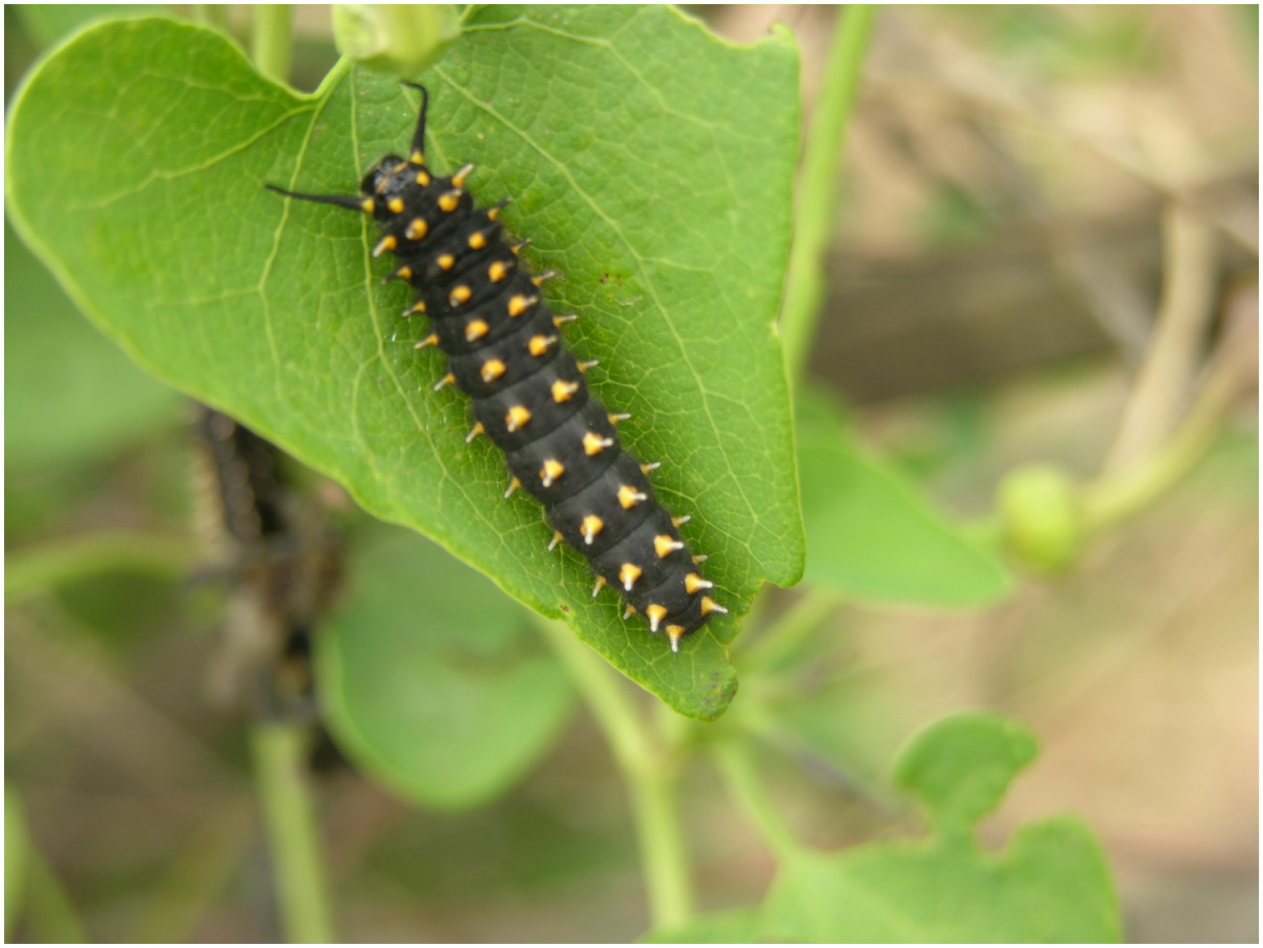
Mature larva of *Sericinus montelus*.

Larvae: The species has five larval instars. Newly hatched larvae have a body length of 3.15mm, the head capsule diameter is 0.4mm; larvae eat fresh leaves or buds. The first and second instar larvae live together; from the third instar onwards they feed independently. After 4–5 instars the body colour of male and female larvae is obviously different. The bodies of males are light brown while those of females are deep brown. The duration of the first generation larvae is 22.3 ±0.8 days (n = 9) days, while for the second and third generation it is 15.9 ±0.2 days (n = 9). The body length of maturing larvae is 23.7 ±1.0 mm (n = 4) (Fig 6).

Pupae: are of medium size with a length of 21.1 ± 0.3 mm (n = 16). The colour of male and female pupae is distinct. Female pupae are deep brown and male pupae are light brown. The pupal stage of the first and second generation lasts for 10.4 ±0.77 days (n = 15) in summer.

Natural enemies: in the field we observed spiders which feed on eggs and capture adults in their webs and ladybirds which feed on the larvae. Loss from predation can reach 71.5% during the larval stage, based on life table analysis.

### Life table

**Fig 7:**
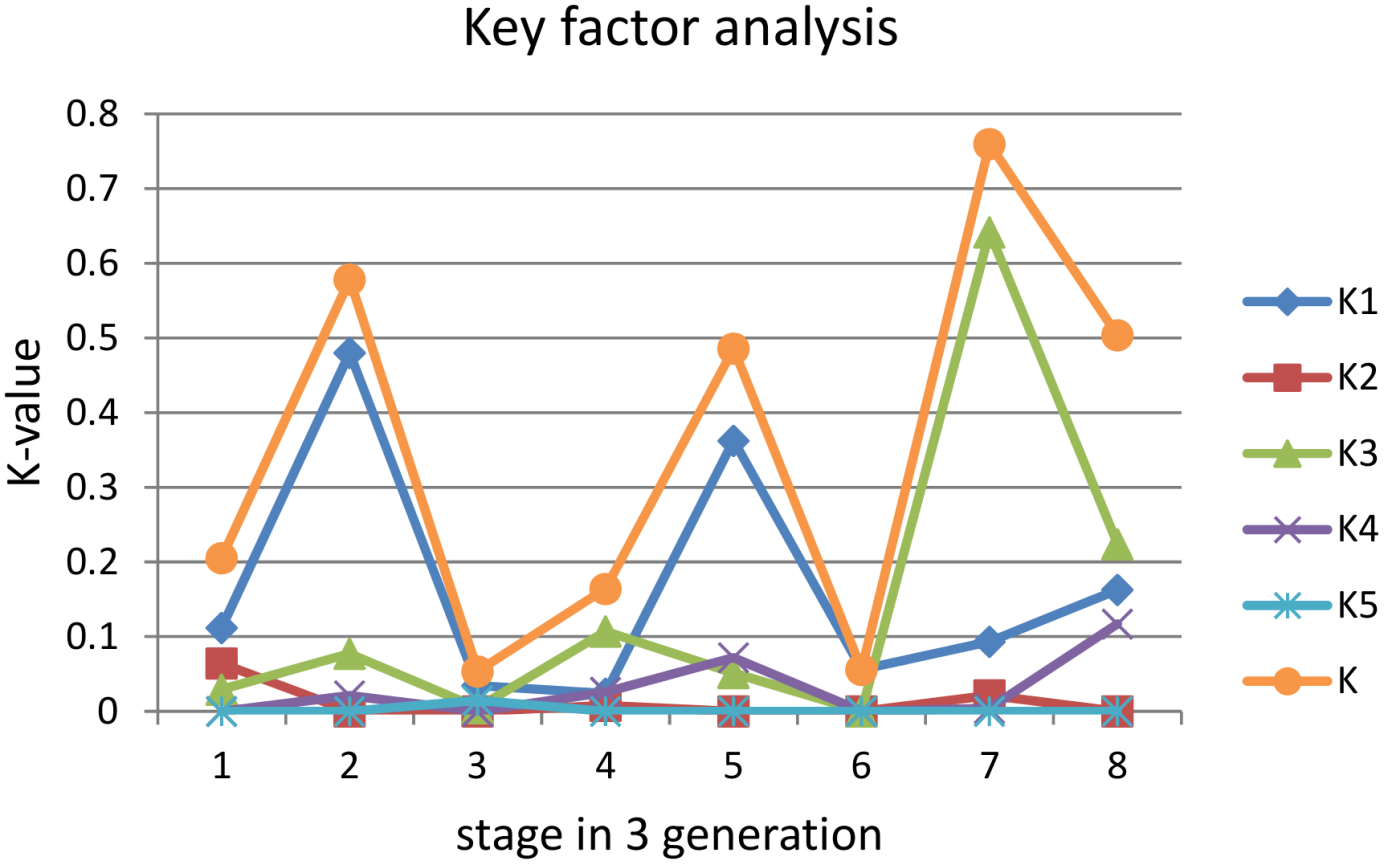
Key factor analysis from life table data of *Sericinus montelus*. K1: natural enemies, K2: hatching/eclosion failure, K3: human activity, K4: pathogens and insolation, K5: death in pupation. x-axis: 1:Egg stage of first generation, 2. First 3 larval instars of first generation, 3: 4^th^ and 5^th^ larval instar of first generation, 4. Egg stage of second generation, 5. First 3 larval instars of second generation, 6. 4^th^ and 5^th^ larval instar of second generation, 7. Egg stage of third generation, 8. First 3 larval instars of third generation.

Derived from the life table (Tables 2 & 3), the key factor of the first and second generation are natural enemies, which cause mortality up to 86% for eggs and 64% for larvae. But in the third generation human activities have been identified as the key factor, which causes mortality of up to 42% for eggs and 75% for larvae (see Tables 2 & 3; Fig 7).

**Table 2:** Stage-specific life table of the three generations of *Sericinus montelus* in 2006, Gaoqiao village.

Generation	Stage	Total number	Cause of mortality	Number of individuals died	Mortality (%)	Survival Rate(%)	K-value
	X	Lx	dxF	(dx)	100qx	100Sx	Log(Lxi/Lxi+1)
First	Eggs	1334		573	42.95	57.05	
		1031	Natural enemies	303	22.71	77.29	K1 = 0.1119
		1151	No hatching	183	13.72	86.28	K2 = 0.0641
		1247	Mowing	87	6.52	93.48	K3 = 0.0293
							K = 0.2053
	1–3 Instar larvae	761		669	87.91.	12.09	
		252	Natural enemies	509	66.89	33.11	K1 = 0.4800
		637	Mowing	124	16.29	83.71	K3 = 0.0772
		725	Pathogens and solar radiation	36	4.73	95.27	K4 = 0.0210
							K = 0.5782
	4–5 Instar larvae	92		11	11.96	88.04	
		85	Natural enemies	7	7.61	92.39	K1 = 0.0344
		91	Mowing	1	1.1	98.9	K3 = 0.0047
		89	Death during pupation	3	3.26	96.74	K5 = 0.0144
							K = 0.0535
	Pupae	22		0	0	100	
Second	Eggs	522		181	34.67	65.33	
		494	Natural enemies	28	5.36	94.64	K1 = 0.0239
		513	Failure to hatch	9	1.72	98.82	K2 = 0.0076
		408	Mowing	114	21.84	78.16	K3 = 0.1070
		492	Pathogens and solar radiation	30	5.75	94.25	K4 = 0.0257
							K = 0.1642
	1–3 Instar larvae	341		283	82.99	7.01	
		148	Natural enemies	193	56.60	43.4	K1 = 0.3625
		303	Mowing	38	11.14	88.86	K3 = 0.0513
		289	Pathogens and solar radiationn	52	15.25	84.75	K4 = 0.0719
							K = 0.4857
	4–5 Instar larvae	58		7	12.07	12.07	
		51	Natural enemies	7	12.07	12.07	K1 = 0.0559
							K = 0.0559
Third	eggs	565		277	49.03	50.97	
		456	Natural enemies	109	19.29	80.71	K1 = 0.0931
		538	Failure to hatch	27	4.78	95.22	K2 = 0.0213
		129	Mowing	136	24.07	75.93	K3 = 0.6415
		560	Pathogens and solar radiation	5	0.88	99.12	K4 = 0.0039
							K = 0.7598
	1–3 Instar larvae	288		274	95.14	4.86	
		198	Natural enemies	90	31.25	68.75	K1 = 0.1627
		172	Mowing	116	40.28	59.72	K3 = 0.2239
		220	Pathogens and solar radiation	68	23.61	76.39	K4 = 0.1170
							K = 0.5030
	4–5 Instar larvae	14		0	0	100	

**Table 3:** Time-specific life table (non-continuous; i.e. each stage is regarded as a new starting point) of three generations of *Sericinus montelus* in 2007 in Gaoqiao Village.

Generation	Stage	Total number	Cause of mortality	Number of individuals Died	Mortality (%)	Survival Rate (%)
	X	Lx	dxF	(dx)	100qx	100Sx
First	eggs	91		91	100	0
			Natural enemies	78	85.71	14.29
			Mowing	13	14.29	85.71
	1–3 Instar larvae	239		205	85.77	14.23
			Natural enemies	114	47.7	52.3
			Pathogens and solar radiation	23	9.62	90.38
			Human activity	68	28.45	71.55
	4–5 Instar larvae	22		0		100
Second	Eggs	144		108	75.0	25.0
			Natural enemies	56	38.89	61.11
			Mowing	52	36.11	63.89
	1–3 Instar larvae	305		279	91.48	8.52
			Natural enemies	119	39.02	60.98
			Pathogens and solar radiation	12	3.93	96.07
			Human activity	148	48.52	51.48
	4–5 Instar larvae	38	Natural enemies	1 1	2.63 2.63	97.37
Third	eggs	156		156	100	0
			Human activity	156	100	0
	1–3 Instar larvae	44		39	88.64	11.36
			Natural enemies	4	9.09	90.9
			Heavy rain, solar radiation	2	4.55	95.45
			Human activity	33	75.0	25.0
	4-5Instar larvae	3		0	0	100

**Fig 8:**
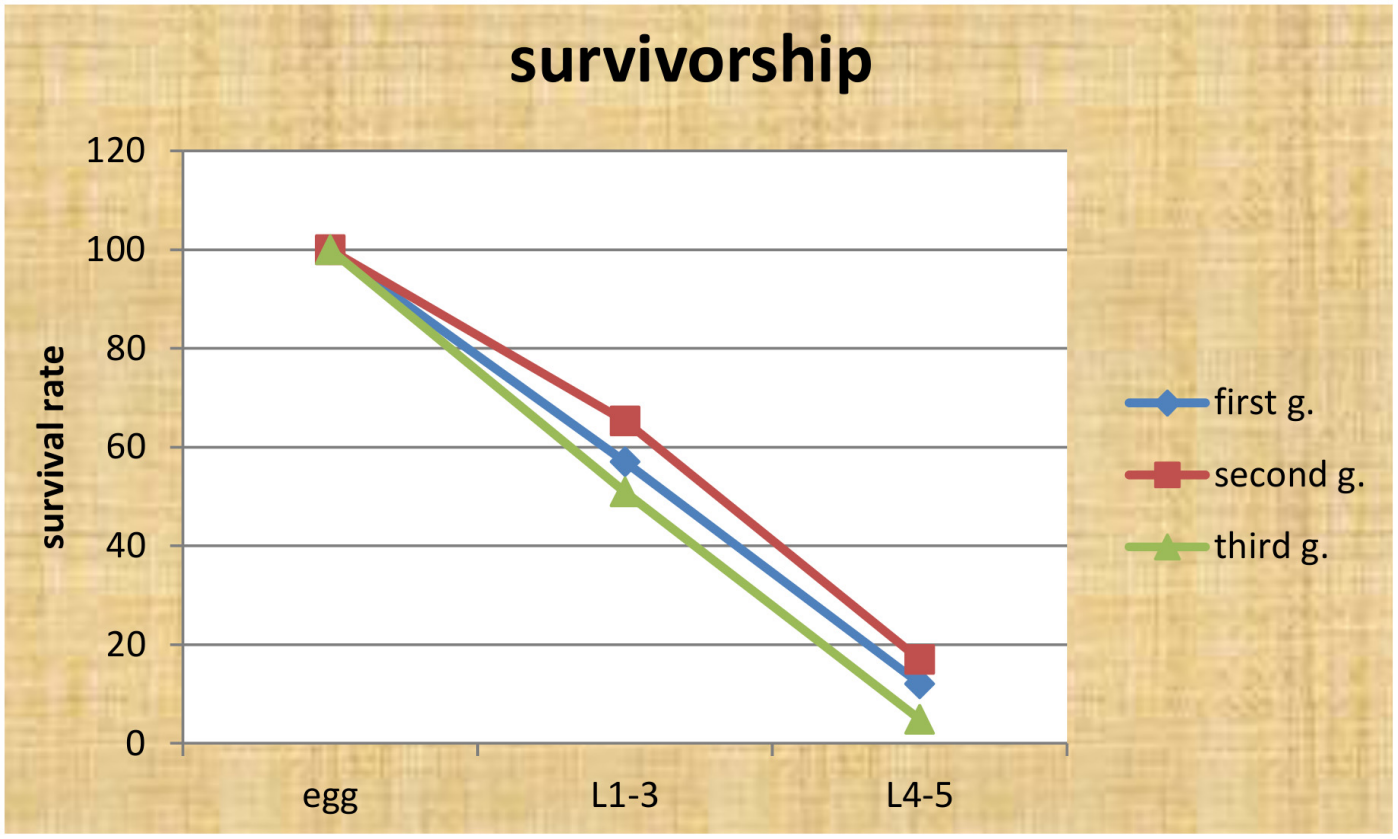
Stage-wise Survival curve of *Sericinus montelus*.

When summarizing the main results of the different life tables (Tables 4 & 5), it becomes obvious that natural enemies are the most important factor, while human activity such as mowing, weeding, grazing, trampling was second, with an average survival rate to old larvae of 11% (Fig 8).

**Table 4:** Comparison of egg and larval mortalities (in %) averaged across 6 generations (Gaoqiao Village).

Stage	Natural enemies	Human activity	Hatching failure	Pathogens& insolation	Death during pupation
eggs	28.66±31.13	33.80±33.91	3.37±5.40	1.10±2.30	
1–3 Instar	41.76± 19.56	36.61±23.48		10.28±7.83	
4–5 Instar	3.72± 5.05	0.18±0.44			0.54±1.33

**Table 5:** Average mortality of eggs and larvae in different generations (%).

Stage	Factor	1	2	3
Eggs	E	54.21	22.13	9.65
	H	10.41	28.97	60.04
Larvae 1–3	E	27.30	47.81	20.17
	H	22.37	29.83	57.64

E: natural enemies; H: human activity

### Population characteristics

For the CMR studies we marked a total of 607 individuals for three generation in 2006 and 2007. Of them only 20 were female but 587 were male. 45 individuals (7.4%) were recaptured. Four individuals (0.66%) were recaptured twice. The recapture rate is related to capture intensity (1 person 4.97%, 2 persons 10.5%).

The observed maximum flight distance of males over their entire lifetime was not more than 700 m (and not more than 400 m per day). 89.3% of individuals moved within a range of 400 m throughout their life. Females were hard to find in the field (only 20 individuals in the three CMR periods). Because after eclosion they mate, lay eggs on the same or next day and then die. The life spans of males have been observed for a maximum of 6 days.

The spatial population structure of *S*. *montelus* shows patchy conditions in Gaoqiao village, composed of three larger and six smaller patches. Assumedly, because of the isolation imposed by villages and mountain ridges, only one male was recaptured from one big patch (Shujiazhuang), which migrated to another patch (in 700m distance). This give some indications that the adults encounter difficulties to migrate between patches (Fig 4), but may also show that field work was not sufficient to detect movements.

The sex ratio based on pupae breeding in the experimental area is 14 females/32 males = 1: 2.29. While from field data based on CMR it is 1: 29.4. These discrepancies in the lab and field data indicate higher detectability for males in the field; quite likely due to sex-specific differences in the activity patterns (females tend to be quite sedentary and very short lived).

The analysis of CMR for two generations in 2006 and one generation in 2007 during the peak flying periods showed that the estimated population size was below 500 individuals for all patches in the capture periods of the second generation in June 2006. It varied quite a lot between slightly less than 10 and around 70 individuals per capture event in the third generation of July/August 2006, and was between 5 and 40 individuals in the second generation in June 2007.

Population dynamics of total population sizes across all patches for three generations from 2006 to 2007 range from 240 to 465 individuals.

### Habitat investigation

Farmland near forest is the preferred habitat of the species. There it occurs along field margins, paths and irrigation ditches, with an average host plant density of 0.55/m^2^ which seems sufficient for the local survival of larvae. The proportion of host plant which has larvae is 11.05% on average, larval density is 0.51 individuals per host plant. But in 7 transects in forests near farmland (400-3000m long and 2m wide), with a forest cover rate of 0.85–0.9, there are no host plants and thus no eggs and larvae (for further details on habitat requirements, counting events and transect investigations see Table 6).

**Table 6:** Distribution of host plants and larvae of *Sericinus montelus* on agricultural land (Aug. 2005; Gaoqiao Forestry Farm).

Sample	Place	Habitat type	Elevation	Length* Width (m*m)	Number of host plants	Density (individ. /m^2^)	N (number) of eggs or larvae		Numbers of host plants with larvae or eggs	Proportion of host plants with larvae or eggs (%)	Larval density (individuals per host plant)
							Eggs	Larvae			
01	F.f. gate	Field bank	1291	100*2	140	0.70	0	62	20	14.29	0.44
02	F.f. gate	Field bank	1293	100*2	135	0.68	0	61	16	11.85	0.45
03	F.f. gate	Field bank	1290	100*2	69	0.35	0	6	5	7.25	0.087
04	F.f. gate	Field bank	1290	100*2	162	0.81	0	7	4	2.47	0.043
05	Guobian	Field bank	1000	100*2	17	0.09	0	10	4	23.53	0.59
06	Guobian	Field bank	1000	100*2	122	0.61	364	7	10	8.20	3.04
07	Xiaeiergou	Forest margin	1300	100*2	69	0.37	0	0	0	0	0
12	Miaozigou	Forest margin	1280	100*2	273	1.36	0	20	9	3.30	0.07
13	Cuijiazhuang	Maize field bank	1283	100*2	74	0.37	0	7	3	4.05	0.09
17	Siheyi Village	Maize field bank	1200	100*2	78	0.39	0	18	10	12.82	0.23
18	Siheyi Village	Maize field bank	1200	100*2	62	0.31	0	33	21	33.87	0.53
Average						0.55				11.05	0.51

Note * 7 transects in forest near farmland, (400*2–3000*2m²); forest cover rate 0.85–0.9, no host plants, no eggs and larvae.

## Discussion

### Main threats for the persistence of *S*. *montelus* populations

*S*. *montelus* in the Xiaolongshan and Taibai Mountain is easily influenced by human agricultural activity. For example, agricultural cultivation, mowing, weeding, trampling and grazing during the egg and larval stages. The host plant is destroyed and then larvae are killed, especially in the second and third generation, when farmers’ agricultural activities are more intense. Additionally, natural enemies cause high mortality in eggs and larval stage.

Agricultural land-use is known as a major factor impacting butterflies across the world [40]. While historically land use has been a driver for increased biodiversity of butterflies in temperate areas, especially in Europe [41,42], the more massive and abrupt changes cause major problems there in recent decades. Quite different to this, land use in most subtropical and tropical areas is a rather new phenomenon which came in much more massively within a short and recent time period, and thus caused massive problems for species in general [43].

After 8 years we went back to our study sites in 2015 and huge land use change was observable. Because of very suitable natural condition for nursery stock, the local government promotes and supports plant nurseries (due to higher economic gains compared to standard crops). Almost all of the farmland in Gaoqiao village was now changed into nursery stock land. Due to pests such as aphis and scale insects, pesticides are more widely used in nursery stock land. The host plants of *S*. *montelus* grow along field margins and the irrigation ditches. The pesticides may easily drift to these host plants and potentially kill the larvae. Thus, although conditions for *S*. *montelus* seemed rather good, no population increase could be observed.

As the adults seem not to be able to disperse for large distances, also the distance of patches on a landscape scale is thus an important component for the survival of the species. In addition the isolation may be enhanced through elements with a barrier effect (e.g. villages and mountain ridges). While connectivity on the landscape scale might be important, we also have to consider the size of single habitat patches. From our CMR data, the observed maximum flight distance over their entire lifetime was not more than 700 m. 89% of individuals moved within a range of 400 m throughout their entire life. So we consider an area within a radius of 400 m as the “home range”, which is equivalent to roughly 0.13 km^2^—an area which might be sufficient at least for the short term survival of a local population of the species. Females can lay more than 100 eggs, theoretically this species has a high reproduction rate, which could lead to large population sizes. However, these were always quite small, but at the same time also quite stable. We think, human agricultural activities, natural enemies and the species’ own characteristics restrict the chances for population increases. On the other hand, eggs and younger larval stages have a high mortality from natural enemies, which might also be an indication of their relevance as food of parasites and predators.

*S*. *montelus* a typical northern China species, distributed at margin area of forest in Xiaolongshan forest region from north to south (see Fig 1). In our research, we observed it is very sensitive on temperature. Taibai village (20 km East of Gaoqiao village) has a 200 m lower elevation than Gaoqiao village and thus a higher temperature which leads to an emergence and egg hatching of two weeks earlier than in Gaoqiao village.

In 2006 around 10% of the supposedly overwintering pupae eclosed in September, possibly because of higher temperature compared to normal years. However, as in late September temperatures are already very low, their life cycle could not be finished which surely led to a higher than normal mortality in this stage. This means that we have to thoroughly watch future developments of the species in the investigation area as climate change (in terms of higher temperatures) might play a role for the future of the species.

### Population maintenance

According to our key factor analysis and species ecological characteristic research, the main factors which restrict population increase are human activities, natural enemies, and short fly distances and life spans of adults. We cannot change the natural factors and species’ own ecological characteristics. So our suggestion is to reduce the damage on host plant, *A*. *contorta*, in agriculture activities, and reduce use of pesticide, so that a minimal amount of *A*. *contorta* is maintained for this species’ survival. Expanding the patch area and an increase of patch connectivity is also useful for population survival.
